# Presynaptic protein synthesis required for NT-3-induced long-term synaptic modulation

**DOI:** 10.1186/1756-6606-4-1

**Published:** 2011-01-07

**Authors:** H Shawn Je, Yuanyuan Ji, Ying Wang, Feng Yang, Wei Wu, Bai Lu

**Affiliations:** 1 Genes, Cognition and Psychosis Program (GCAP), National Institute of Mental Health, NIH, Bethesda, MD 20892, U.S.A; 2 Program in Neuroscience and Behavioural Disorders, DUKE-NUS Graduate Medical School, 169857, Singapore; 3 GlaxoSmithKline, R&D China, Pudong, Shanghai, 201203, China; 4 School of Life Sciences, Tsinghua University, Beijing 100084, China; 5 Protein Science Laboratory of the Ministry of Education, Tsinghua University, Beijing 100084, China

## Abstract

**Background:**

Neurotrophins elicit both acute and long-term modulation of synaptic transmission and plasticity. Previously, we demonstrated that the long-term synaptic modulation requires the endocytosis of neurotrophin-receptor complex, the activation of PI3K and Akt, and mTOR mediated protein synthesis. However, it is unclear whether the long-term synaptic modulation by neurotrophins depends on protein synthesis in pre- or post-synaptic cells.

**Results:**

Here we have developed an inducible protein translation blocker, in which the kinase domain of protein kinase R (PKR) is fused with bacterial gyrase B domain (GyrB-PKR), which could be dimerized upon treatment with a cell permeable drug, coumermycin. By genetically targeting GyrB-PKR to specific cell types, we show that NT-3 induced long-term synaptic modulation requires presynaptic, but not postsynaptic protein synthesis.

**Conclusions:**

Our results provide mechanistic insights into the cell-specific requirement for protein synthesis in the long-term synaptic modulation by neurotrophins. The GyrB-PKR system may be useful tool to study protein synthesis in a cell-specific manner.

## Background

Synaptic plasticity, or activity-dependent morphological and functional modification of synaptic connections, is the dominant underlying mechanism for brain function [1]. Recently, neurotrophins, a family of structurally and functionally related proteins, that include nerve growth factor (NGF), brain derived neurotrophic factor (BDNF), neurotrophin-3 (NT-3), and neurotrophin-4/5(NT-4/5), have emerged as major modulators involved in synaptic plasticity [2-4]. Similar to synaptic plasticity, synaptic effects of neurotrophins can be divided into two temporally distinct modes: the acute effect occurring within seconds or minutes upon a neurotrophin exposure, and the long-term effect taking hours and days to accomplish [5-7]. Previously, we identified that the acute and long-term effects of NT-3 are operated by distinct molecular and cellular mechanisms by using *Xenopus *cultured neuromuscular synapse [6,8]. Compared to acute effects, NT-3 mediated long-term synapse modulation requires endocytosis of NT-3-TrkC (a cognate receptor for NT-3) complex, activation of Akt, a major downstream kinase of PI3K pathway, and mTOR dependent protein synthesis [6].

The requirement for protein synthesis assumes that NT-3 can trigger protein synthesis which can occur in presynaptic neurons or postsynaptic muscle cells [9]. Because conventional pharmacology cannot inhibit protein synthesis in a cell-type specific manner, we developed and utilized an inducible protein translation blocker that can be genetically targeted to specific cells to further investigate whether NT-3 induced long-term synaptic modulation requires either presynaptic or postsynaptic protein synthesis [10]. Our protein synthesis inhibitor system utilizes the double-stranded (ds) RNA-dependent protein kinase (PKR), which reversibly phosphorylates the α subunit of eukaryotic initiation factor-2 (eIF2α) to control protein synthesis in eukaryotic cells [11]. The kinase activity of PKR is very low at rest, but is significantly induced upon binding of its dsRNA-binding domains to dsRNAs during viral infection, leading to dimerization, autophosphorylation, activation of the kinase, and eventual blockade of general mRNA translation [12]. To establish an inducible system, we utilized bacterial gyrase B domain, which could be dimerized upon treatment with a cell permeable drug, coumermycin [13]. By using this unique system that allows specific inhibition of general mRNA translation only on expressing cells, we show that NT-3 induced long-term synaptic modulation requires presynaptic, but not postsynaptic protein synthesis. Taken together, these results suggest general principles that govern long-term regulation of synapses by neurotrophins.

## Results

### GyrB-PKR, an inducible molecular system to block protein synthesis

**Figure 1:**
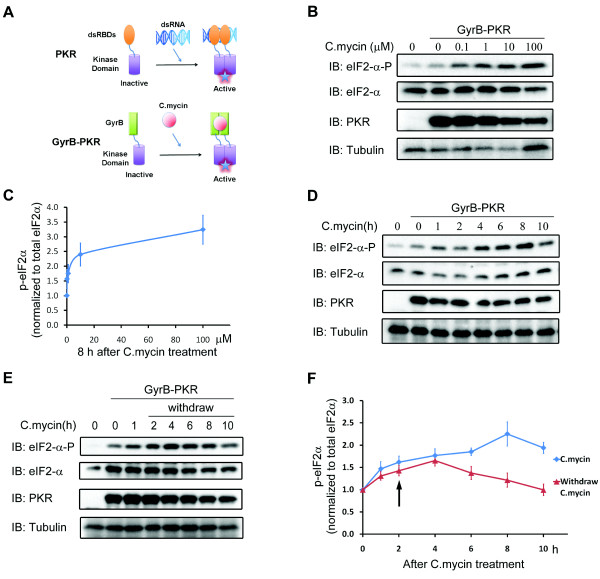
**Phosphorylation of eIF2α upon coumermycin-induced GyrB-PKR dimerization**. (A) Schematic diagrams showing the fusion of PKR kinase domain with coumermycin-binding domain of GyrB. Application of coumermycin (C. mycin) induces the dimerization of the PKR fusion proteins and activates PKR, triggering eIF2α phosphorylation and subsequent de novo protein synthesis inhibition. (B) Representative blots showing C.mycin-induced phosphorylation of eIF2α on Ser51 in *Xenopus *embryos expressing GyrB-PKR. Embryos were treated with various concentrations of coumermycin for 8 hours, harvested and lysed. Western blotting was performed using specific antibodies as indicated. The blots were also probed with anti-eIF2α and anti-tubulin antibody for loading controls. (C) Quantification of eIF2α phosphorylation with different C.mycin concentrations. (D) Time course of eIF2α phosphorylation induced by 1 μM Coumermycin. (E) Time course of eIF2α phosphorylation upon 1 μM C.mycin treatment and withdrawal. Embryos were treated with Coumermycin for 2 hours and washed with culture medium without C.mycin (F) Quantification of eIF2α phosphorylation with or without C.mycin at different time points. Arrow indicated the time point of withdrawing C.mycin. Note the eIF2α phosphorylation level goes back to the baseline within 8 hours. Multiple blots were quantified (N = 6), and eIF2α-P signals at various time points were normalized to that at "0" hour.

Previously, we found that the rapamycin (200 nM), a specific inhibitor for mTOR, blocked NT-3 induced long-term synapse modulation [6]. Pharmacological inhibitors may elicit side effects in addition to its inhibition of protein synthesis [14,15]. It is also unclear whether rapamycin acts pre- or postsynaptically. Here we attempted to develop a genetic approach to examine the importance of protein synthesis in NT-3-induced synaptic modulation. The dimerization of PKR kinase domain has been shown to be both necessary and sufficient to activate its kinase function [13], which could suppress protein synthesis by phosphorylating eIF2α, leading to the dissociation of eIF2-tRNA-40 S complex [11]. We replaced dsRNA-binding domain of PKR with *E. coli *protein gyrase B, which could be dimerized upon exposure to the cell-permeable ligand coumermycin [16]. This fusion protein GyrB-PKR should therefore in theory confer inducible and reversible inhibition of protein synthesis upon treatment with coumermycin (Figure 1A).

To determine whether coumermycin truly induced dimerization and activation of GyrB-PKR, we expressed GyrB-PKR in developing *Xenopus *embryos by blastomere injection techniques [17]. Western blot analysis was used to monitor the expression of GyrB-PKR and phosphorylation of eIF2α, a direct downstream target of PKR, upon treatment with coumermycin at various concentrations and durations. Addition of 0.1 μM coumermycin caused eIF2α phosphorylation (Figure 1B). The half-maximum response (EC_50_) value for coumermycin-induced eIF2α phosphorylation was 1 μM, which was measured 8 hours after drug treatment (Figure 1C). Coumermycin treatment led to a robust eIF2α phosphorylation as early as 5 min, which lasted more than 10 hours (Figure 1D and 1F). Moreover, when coumermycin was removed 2 hours after its application, the eIF2α phosphorylation began to decline at 4-hour and reached baseline levels at 10-hour (Figure 1E and 1F). Taken together, these experiments indicate that the expression of GyrB-PKR results in inducible and reversible phosphorylation of eIF2α upon coumermycin treatment.

**Figure 2:**
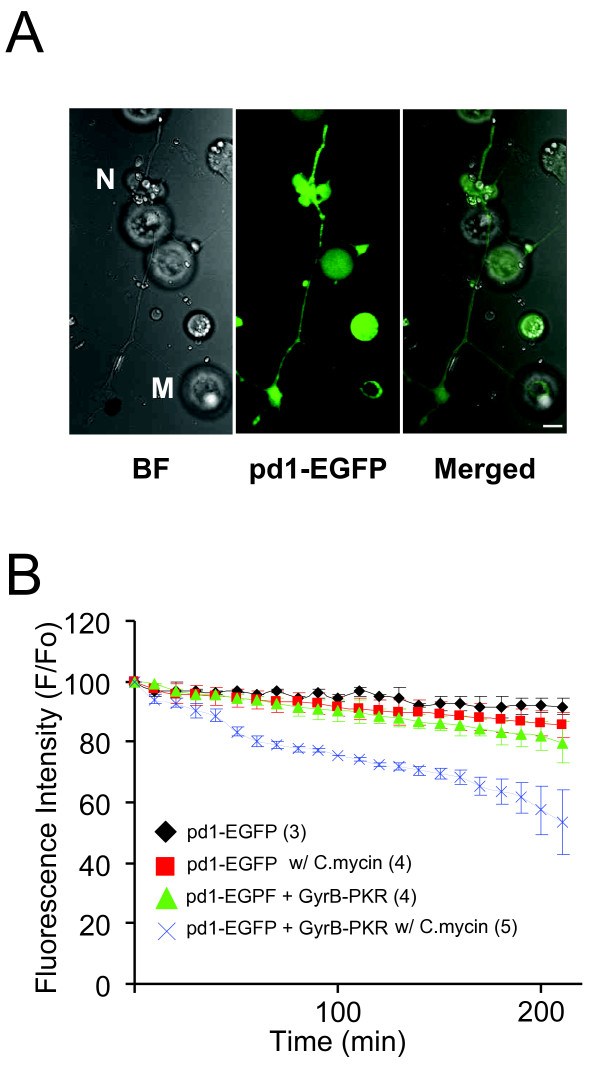
**Inhibition of protein translation in spinal neurons by C.mycin -induced GyrB-PKR dimerization**. (A) Expression of the pd1-EGFP in *Xenopus *spinal neurons. Bright field, fluorescent, and merged images (left, middle, right, respectively) show a spinal neuron and muscle cells expressing pd1-EGFP. N: neuron; M: muscle cell. Bar, 10 μm. (B) Quantification of fluorescence change on neurons. Pd1-EGFP was expressed either alone or together with GyrB-PKR in *Xenopus *spinal neurons, using embryo injection techniques. C.mycin was applied to the culture dish at time "0", and pd1-EGFP fluorescence was monitored over time. The number of experiments performed was indicated in the legend.

Next, we investigated whether the dimerization and subsequent activation of PKR inhibits new protein synthesis. Due to limited number of cells available in these nerve-muscle co-cultures, it was not feasible to directly measure protein synthesis using conventional approaches, such as ^3^H-leucine incorporation. Thus, we utilized a destabilized green fluorescence protein (pd1-EGFP, half live = 1 hour) whose fluorescence fades if protein synthesis is blocked. In pd1-EGFP, the residues 422-461 of mouse ornithine decarboxylase (MODC) were fused to the C terminus of EGFP to enable a rapid protein degradation and turnover [18]. Therefore, by measuring GFP fluorescence change, we could monitor steady-state levels of GFP proteins, which should correlate with the degree of general protein synthesis. When pd1-EGFP was expressed in spinal neurons by embryo injection (Figure 2A), treatment of the cultures with the general protein synthesis inhibitor rapamycin (200 nM) or cyclohexamide (60 μM) for 1 hour greatly reduced fluorescence intensity as a consequence of the inhibition of new EGFP synthesis, which indicated the feasibility of monitoring protein synthesis using this assay (data not shown) [19].

To determine whether coumermycin treatment inhibits protein synthesis in cultured spinal neurons, we expressed pd1-EGFP with or without Gyr-PKR in *Xenopus *spinal neurons and monitored the changes in fluorescent intensity upon coumermycin treatment. Indeed, coumermycin treatment reduced the GFP fluorescent intensity by 45% in spinal neurons only when pd1-EGFP co-expressed with GyrB-PKR (Figure 2B). Taken together, these results demonstrate that coumermycin induced dimerization of PKR effectively phosphorylates eIF2α and subsequently blocks new protein synthesis.

### Presynaptic protein synthesis in NT-3-induced synaptic modulation

**Figure 3:**
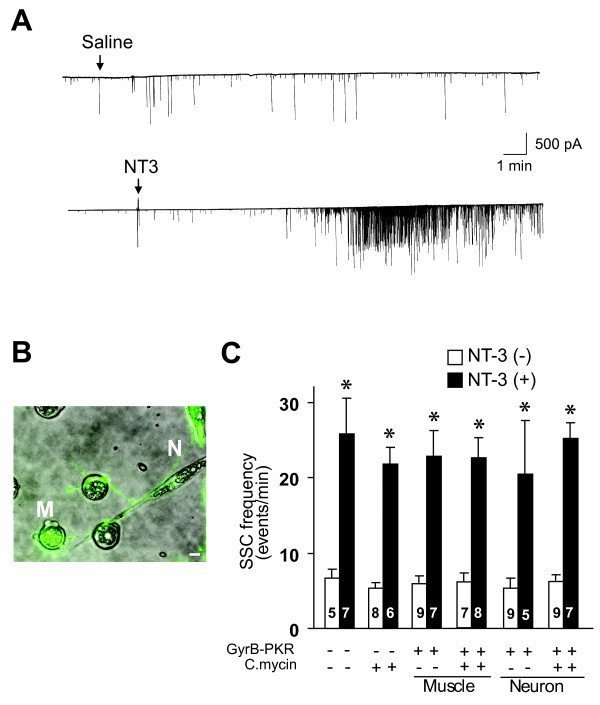
**Normal acute synaptic potentiation by NT-3 in GyrB-PKR activated neuromuscular synapses**. (A) Sample recordings showing acute synaptic potentiation by NT-3. NT-3 (50 ng/ml) was applied directly to 1-day-old nerve-muscle co-culture. The frequency of spontaneous synaptic currents (SSCs) was used to monitor changes in synaptic efficacy. (B) A sample image showing neuromuscular synapses in which muscle cells are innervated by a GFP expressing spinal neuron. N: neuron axon (neuron body is out of this image); M: muscle cell. Bar, 10 μm. (C) Summary of acute effect of NT-3 on synapses without GyrB-PKR expression, and synapses with presynaptic or postsynaptic expression of GyrB-PKR. Each data point represents SSCs frequency (averaged from 30 min of recording) from a single synapse before C.mycin and after NT-3 application. Note that the application of coumermycin did not affect the baseline of recording. The number associated with each column represents the number of cells analyzed. Data are presented as the mean ± the SEM.

At the *Xenopus *neuromuscular synapses, application of exogenous NT-3 at a high concentration (50 ng/ml) induces a rapid potentiation of synaptic transmission within 5 min [20,21], whereas long-term treatment with a lower concentration of NT-3 (5 ng/ml; 2 days) facilitates physiological and morphological maturation of the synapses [6,8,17]. We recorded spontaneous synaptic currents (SSCs) in 1-d old nerve-muscle co-culture using whole-cell voltage clamp recording techniques. As reported, acute application of NT-3 elicited a marked increase in transmitter release in neurons (Figure 3A, bottom). The same treatment in the presence of coumermycin didn't affect NT-3 mediated acute effect, indicating that coumermycin itself did not affect synaptic transmission (Figure 3C, columns 3&4).

The embryo injection technique allows selective expression of GyrB-PKR in either presynaptic motor neurons or postsynaptic myocytes, as indicated by co-expressed GFP fluorescence, at neuromuscular synapses in the nerve-muscle co-culture (Figure 3B). Using this system, we tested whether activation of GyrB-PKR either presynaptically or postsynaptically alters the NT-3 effect. When GyrB-PKR was expressed in the postsynaptic muscle cells, application of NT-3 in the presence of coumermycin had no effect on the acute synaptic potentiation induced by NT-3 (Figure 3C, columns 5-8). Similarly, the expression of GyrB-PKR in presynaptic motor neurons also failed to alter the NT-3 effect in coumermycin treated cultures (Figure 3C, columns 9-12). These results together suggest that the acute synaptic potentiation by NT-3 does not require protein synthesis.

**Figure 4:**
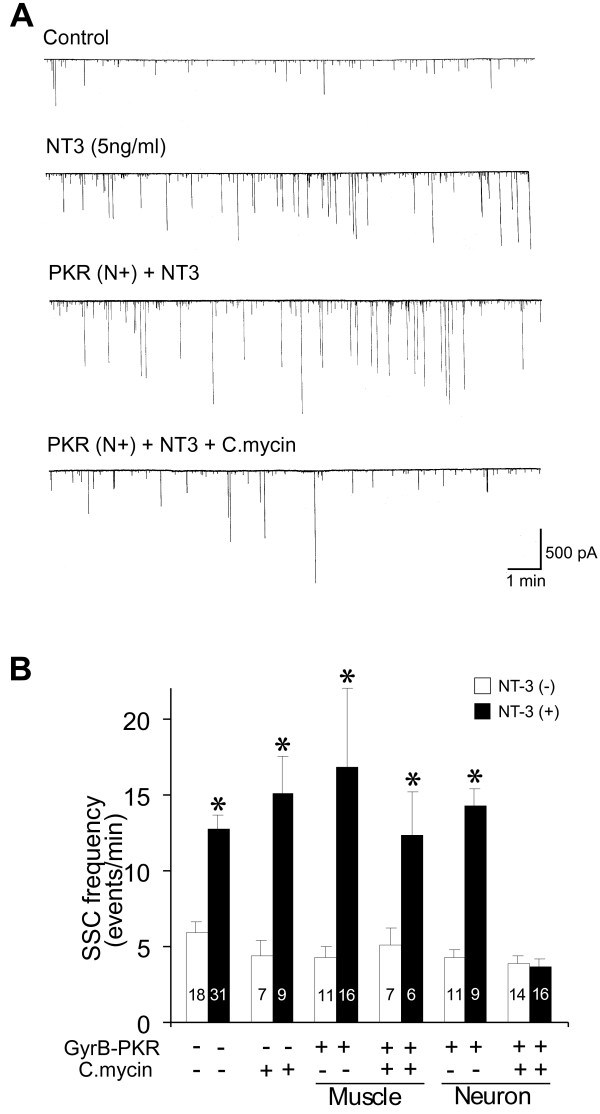
**Blockade of the long-term synaptic effect of NT-3 by C.mycin -induced PKR activation**. (A) Sample recordings showing long-term synaptic potentiation by NT-3. The *Xenopus *nerve-muscle co-cultures were grown in the presence or absence of NT-3 (5 ng/ml) for 2 days. Expression of GyrB-PKR alone did not prevent NT-3-mediated long-term synaptic potentiation. However, application of C.mycin to synapses expressing GyrB-PKR presynaptically completely prevented long-term synaptic potentiation by NT-3. (B) Summary of long-term effect on NT-3 on synapses without GyrB-PKR expression, and synapses with pre- or post-syanptic expression of GyrB-PKR. Each data point represents SSCs frequency (averaged from 30 min of recording) form a single synapse with or without coumermycin treatment. Note that application of C.mycin alone could not attenuate the increase in SSC frequency by NT-3 chronic treatment. Application of C.mycin to synapses expression GyrB-PKR presynaptically, but not postsynaptically, completely prevented long-term synaptic effect of NT-3. The number associated with each column represents the number of cells analyzed. Data are presented as the mean ± the SEM.

Next, to determine whether pre- or post-synaptic protein synthesis is necessary for NT-3 mediated long-term synaptic modulation, we expressed GyrB-PKR in either spinal neurons or myocytes using the same embryo injection techniques described above. Cultures were incubated with NT-3 (5 ng/ml) for 2 days with or without coumermycin as indicated (Figure 4A). At naive synapses, coumermycin treatment did not affect basal synaptic transmission nor prevent the long-term potentiating effect of NT-3 (Figure 4B, columns 3&4). Expression of GyrB-PKR in either presynaptic spinal neurons (Figure 4B, columns 5&6) or postsynaptic muscle cells (Figure 4B, columns 9&10) without coumermycin treatment did not alter the long-term effect of NT-3. Intriguingly, coumermycin treatment completely blocked the long-term effect of NT-3 in synapses made by spinal neurons expressing GyrB-PKR (Figure 4B, columns 11&12). However, the same treatment was ineffective if GyrB-PKR was expressed in postsynaptic myocytes (Figure 4B, columns 7&8). Taken together, these results suggest that protein synthesis in the presynaptic spinal neurons but not postsynaptic muscle cells is critical for NT-3 mediated long-term synaptic modulation at neuromuscular synapses.

## Discussion

### Targeting protein synthesis inhibition to specific cells

We have previously described an inducible PKR system that is based on dimerization of FKPB-PKR induced by the synthetic ligand AP20187 [10,22]. Here we report a similar system based on GyrB-PKR induced by coumermycin. Both systems have a major advantage over the conventional pharmacological inhibition of protein synthesis: genetically targeting to a specific cell population. This is particularly valuable in heterogeneous system in which cell-cell interaction is prominent, such as pre- and postsynaptic interactions in the nervous system. The GyrB-PKR system is attractive in several ways. First, coumermycin is an antibiotic that is not toxic to vertebrate cells. In our hands, incubation with coumermycin at 1 μM for two days showed no obvious adversary effect to the nerve-muscle cultures (unpublished observations). Second, in the GyrB-PKR fusion construct, the dsRBD is removed and replaced it with GyrB, a bacterial protein that dimerizes upon binding to coumermycin. This modification prevents non-specific activation of PKR by other agents. Third, the only clearly verified substrate of PKR is the eukaryotic translation initiation factor eIF2α [13]. Phosphorylation of Ser51 on eIF2α converts it from a substrate to a competitive inhibitor of the guanine-nucleotide exchange factor eIF2B, blocking general mRNA translation. Activation of PKR therefore represents a specific inhibition of protein synthesis with relatively few side effects. Finally, using embryo injection techniques, we show that the GyrB-PKR system is very useful in selective inhibition of protein synthesis in pre- or postsynaptic cells. Taken together, the GyrB-PKR system offers an alternative way to inducibly and reversibly block protein synthesis in the targeted cells, allowing applications in situations when AP20187 could not be used.

### Pre- and postsynaptic protein synthesis in synaptic modulation

Compelling evidence suggests that one of the fundamental differences between acute and long-term synaptic modulation by neurotrophins is the requirement for protein synthesis. Acute potentiation of synaptic transmission by NT-3 is completely insensitive to translation inhibitors such as anisomycin and cycloheximide [23]. In contrast, the long-term form of NT-3 mediated synaptic modulation, including both structural (synaptic varicosity) and functional (synaptic currents) changes, requires protein synthesis. Bath application of rapamycin, a protein synthesis blocker by targeting mTOR, completely reversed the morphological and physiological changes induced by long-term exposure to NT-3 [6]. The major contribution of the present study is to demonstrate that at the developing neuromuscular synapses, it is the protein synthesis in the presynaptic motor neurons, but not in postsynaptic muscle cells, that mediates NT-3 induced long-term synaptic modulation. We show that coumermycin can effectively block the NT-3 effects only when the GyrB-PKR is selectively expressed in the pre- but not in postsynaptic cells. This finding indicates that NT-3 stimulates protein synthesis in presynaptic neurons, supplying the necessary proteins to enhance synaptic functions. It remains to be investigated whether the increase in protein synthesis occurs at the motor neuron soma, or in the motor axons or terminals. It should be note that protein synthesis in the presynaptic axon was reported to be involved in activity-dependent synaptic plasticity in sensory motor synapse in *Aplysia *and mature crayfish neuromuscular junctions [24,25].

Similar to neurotrophin-induced synaptic potentiation at the neuromuscular synapses, late-phase long-term potentiation (L-LTP) at the hippocampal CA1 synapses also depends on protein synthesis [5,26]. By injecting FKBP-PKR-expressing virus into CA1, but not CA3, of hippocampus *in vivo*, our previous study demonstrated that postsynaptic, but not presynaptic, inhibition of protein synthesis blocks L-LTP [10]. These results suggest that at the CA1 synapses in the hippocampus, protein synthesis in the postsynaptic CA1 neurons, rather than presynaptic CA3 neurons, is critical in maintaining L-LTP [10]. Thus, for long-term synaptic modulation, there is no set rule for the requirement of protein synthesis in pre- or postsynaptic site.

## Conclusion

We developed an inducible protein synthesis blocker that can be genetically targeted to specific types of cells. By using this novel molecular tool, we have identified that presynaptic protein synthesis is crucial for NT-3-mediated long-term synaptic modulation in *Xenopus *neuromuscular synapses. Our findings elucidate mechanistic insights into the cell-specific requirement for protein synthesis in the long-term synaptic modulation by neurotrophins.

## Methods

### DNA constructs, *Xenopus *embryo injection, nerve-muscle co-culture and whole-cell patch clamp recording

GyrB-PKR construct, which contains a bacterial gene GyrB fused with the kinase domain of PKR (GyrB-PKR, Figure 1A), was described previously [13]. Capped GyrB-PKR mRNAs were synthesized using mMessage machine (Ambion), mixed with GFP mRNA (1 mg/ml) in a 1:1 ratio, and injected into one blastomere at the 2- or 4-cell stage embryos using the Picospitzer pressure ejector as described [6].

Nerve-muscle cultures were prepared one day after injection [6]. Briefly, neural tubes and associated myotomal tissues of *Xenopus *embryos at stage 20 were dissociated in Ca^2+^-Mg^2+^-free medium (58.2 mM NaCl, 0.7 mM KCl, and 0.3 mM EDTA, pH 7.4) for 15-20 min. Cells were plated on clean glass coverslips, and grown in the presence or absence of NT-3 (5 ng/ml, gift from Regeneron Phamaceuticals) for 2 days at room temperature. Coumermycin, which induces GyrB-PKR dimerization, was added 1 hour before NT-3 treatment. The culture medium consisted (vol/vol) of 50% L-15 medium, 1% fetal calf serum and 49% Ringer's solution (117.6 mM NaCl, 2 mM CaCl_2_, 2.5 mM KCl, 10 mM HEPES, pH 7.6).

Synaptic currents were recorded from innervated muscle cells in 1 or 2-day old cultures by the whole-cell patch clamp recording in culture medium at room temperature [8]. The internal pipette solution contained 150 mM KCl, 1 mM NaCl, 1 mM MgCl_2 _and 10 mM HEPES buffer (pH 7.2). The membrane potentials of the muscle cells recorded were generally in the range of -55 to -75 mV and were voltage clamped at -70 mV. All data were collected by an Axonpatch 200B patch clamp amplifier (Molecular Devices), with a current signal filter set at 3 kHz. The frequency of spontaneous synaptic currents (SSCs) was defined as the number of SSC events per minutes. The frequency and amplitude of SSCs were analyzed using Clampfit software (Molecular Devices). Pipette and membrane capacitance and serial resistance were compensated.

### Western blot analysis

Western blotting was performed as described [8]. *Xenopus embryos *at stage 20-22 were quickly homogenized in the extraction buffer (100 mM NaCl, 50 mM Tris-HCl, pH7.5, 1% NP-40, 2 mM PMSF, 1 mg/ml aprotinin, 1 mg/ml leupeptin, 1 mg/ml pepstatin A, 2 M Na_3_VO_4_) and subsequently sonicated. The insoluble pellet after high-speed centrifugation was discarded and the resulting supernatants were transferred to fresh tubes containing 300 ml freon (1,1,2-trichlorotrifluoroethane)(Sigma), vortexed for 1 min, incubated on ice for 5 minutes, and subsequently centrifuged to remove yolk protein. Next, protein concentrations were determined using the Bradford protein assay kit (BioRad). Proteins were separated by SDS-polyacrylamide electrophoresis, and blotted onto Immobilon-P membrane (Millipore) with Semi-dry gel transfer apparatus (BioRad). The blots were incubated overnight at 4°C with primary antibodies including anti-PKR (1:1000) (Cell Signaling Technology), anti-phosphorylated form of eIF2α (1:1000) (Assay designs), anti-tubulin (Covance), and anti-eIF2α (Cell Signaling). Next, blots were incubated with secondary antibodies conjugated with HRP (Jackson Immunoresearch) and signals were detected by chemiluminescence kit (GE healthcare).

### Protein synthesis inhibition assay

Destabilized EGFP vector (pd1EGFP-N1 from clontech) was used to monitor new protein synthesis in *Xenopus *spinal neurons. In the pd1EGFP, residues 422-461 of mouse ornithine decarboxylase (MODC) were fused to the C terminus of EGFP and this region of MODC contains a PEST amino acid sequence that targets the protein for degradation, resulting in rapid protein turnover. This PEST amino acid sequence of MODC is highly conserved in *Xenopus*, mice and human. It is correlated with most short-lived proteins [27]. pd1EGFP has a half-life of approximately one hour, as measured by fluorescence intensity of cells treated with the protein synthesis inhibitor cycloheximide [18]. pd1EGFP and/or GyrB-PKR were expressed in *Xenopus *spinal neurons by embryo injection. Images were collected with 40× objective lens (NA 1.0) on a fluorescence microscopy. Fluorescence intensity from a small region of interest (ROI, 6 pixels by 6 pixels, or 1.0 × 1.0 μm) on a single neuronal soma was measured and analyzed. The pd1EGFP associated fluorescence was calculated by subtracting the fluorescence intensity in background (cell-free) area from the averaged intensity of three different ROIs on neuronal soma. ROIs were initially positioned by eye and corrected for the center of mass of each soma by an automated script in IPLab (Scanalytics). Before drug treatment, average intensities from 3 time frames were considered as an initial fluorescence level. Next, using time-lapse microscopy, images are collected and the fluorescence intensity of each frame was recorded. Fluorescence puncta in one neuron were pooled and averaged. Student t-test was used to analyze average intensity between groups.

## List of abbreviations

(BDNF): Brain derived neurotrophic factor; (C.mycin): coumermycin; (GyrB): Gyrase B; (eIF2): eukaryotic initiation factor-2; (LTP): long-term potentiation; (MODC): mouse ornithine decarboxylase; (NGF): nerve growth factor; (NT-3): neurotrophin-3; (NT-4/5): neurotrophin-4/5; (PI3K): phosphatidylinositol 3-kinase; (PKR): double strand RNA dependent protein kinase; (SSCs): spontaneous synaptic currents .

## Competing interests

The authors declare that they have no competing interests.

## Authors' contributions

HSJ, YW, and FY performed experiments. HSJ and YJ designed experiments and analyzed data. WW participated in the design and coordination of the study. HSJ, YJ, and BL wrote the manuscript. All authors read and approved the final version of a manuscript.
